# Cost-effectiveness of oral alitretinoin in patients with severe chronic hand eczema - a long-term analysis from a Swiss perspective

**DOI:** 10.1186/1471-5945-10-4

**Published:** 2010-06-25

**Authors:** Patricia R Blank, Armin A Blank, Thomas D Szucs

**Affiliations:** 1 Institute of Social and Preventive Medicine, University of Zurich, Hirschengraben 84, 8001 Zurich, Switzerland; 2 Dermatology FMH, Schifflaende 24, 8001 Zurich, Switzerland; 3 ECPM Institute of Pharmaceutical Medicine, University of Basel, Klingelbergstrasse 61, 4056 Basel, Switzerland

## Abstract

**Background:**

The impact on patients suffering from chronic hand eczema (CHE) is enormous, as no licensed systemic treatment option with proven efficacy for CHE is available. Alitretinoin is a novel agent which showed high clinical efficacy in patients with severe, refractory CHE. We assessed the cost-effectiveness of alitretinoin for CHE patient treatment from a Swiss third party payer perspective. A further objective of this study was to determine the burden of disease in Switzerland.

****Methods**:**

A long-term Markov cohort simulation model was used to estimate direct medical costs (€) and clinical effectiveness (quality adjusted life years, QALYs) of treating severe CHE patients with alitretinoin. Comparison was against the standard treatment of supportive care (optimised emollient therapy). Information on response rates were derived from a randomized controlled clinical trial. Costs were considered from the perspective of the Swiss health system. Swiss epidemiological data was derived from official Swiss Statistic institutions.

****Results**:**

Annual costs of alitretinoin treatment accounted for €2'212. After a time horizon of 22.4 years, average remaining long-term costs accounted for €42'208 or €38'795 in the alitretinoin and the standard treatment arm, respectively. Compared with the standard therapy, the addition of alitretinoin yielded an average gain of 0.230 QALYs at the end of the simulation. Accordingly, the incremental cost-effectiveness ratio resulted in €14'816/QALY gained. These results were robust to changes in key model assumptions.

****Conclusion**:**

The therapy for CHE patients is currently insufficient. In our long-term model we identified the treatment with alitretinoin as a cost-effective alternative for the therapy of CHE patients in Switzerland.

## Background

Hand eczema is a widespread dermatological condition associated with a chronic course and poor healing rates [1]. Particularly in severe patients, the response to the treatment is commonly very bad. Despite the currently available therapies, the illness leads frequently to a chronic course [1,2]. It is well known that patient's quality of life is affected negatively by restricted function of the hands, severe psychological strain and also economical implications [3-5].

The prevalence of patients with hand eczema is estimated at 7% to 12% in the general population [5,6]. However, the fraction of severe chronic hand eczema (CHE) among hand eczema patients is assumed to be 5-7%, whereas 2-4% are estimated to be refractory to topical steroids[7]. Given that epidemiological data on CHE is scarce, it is difficult to assess an exact figure of the frequency of work-related CHE [6,8-10].

Mild eczema patients are mostly treated with emollients and topical corticosteroids. On the other hand, only limited treatment agents for severe chronic hand eczema refractory to the standard therapy with topical steroids are available including off-label used agents. Despite the use of these treatments, the efficacy of these treatments remains questionable. Hence, there is a strong need for a novel CHE therapy.

In 2008, alitretinoin, a 9-cis retinoic acid was firstly registered in Germany for use in patients with severe CHE refractory to potent topical corticosteroids [11]. The agent was shown to be highly effective in several clinical trials including the BACH-trial a randomized, double blind, placebo-controlled study in patients with CHE. The anti-inflammatory and immunomodulatory properties of alitretinoin induce high response rates with a favourable safety-profile over a long-term management of the treatment [12-14]. Since 2009, alitretinoin (Toctino^®^, Basilea Pharmaceutica International AG, Basel) has been approved for severe, refractory CHE in Switzerland.

Economic evaluations are essential key implications for health policy and decision makers[15]. Nevertheless, due to the lack of data on costs associated with CHE, only few international studies have measured the economic burden of disease. Some studies exist which reported possible change or even lost of employment in patients with work-related hand eczema [16,17]. The annual costs for work-related CHE was estimated at €9'000[5]. Hence, the health economic impact of disease may be considerable, but data are urgently required to enlighten this issue.

This health economic study aimed to assess the cost-effectiveness of oral alitretinoin in patients with severe CHE refractory to potent topical corticosteroid. The perspective was from the Swiss health care system. In addition to this, this study determined the burden of disease in Switzerland.

## Methods

### Model design

A Markov decision model was developed to estimate the cost and effectiveness of alitretinoin in patients with severe chronic hand eczema (CHE). The model was built using Microsoft Excel (MME Europe, Milano, Italy). Two strategies were studied: treatment with oral alitretinoin or supportive care (optimised emollient therapy). The clinical data and the treatment protocol stem from two double-blind, randomised, placebo-controlled clinical studies (BACH) described by Ruzicka et al [12-14] and a Swiss dermatology opinion leader (Dr. A. Blank, Zurich, Switzerland). A hypothetical cohort of 1'000 patients was simulated with the same endpoints, treatments used, therapy duration, entry criteria and demographic characteristics as documented in these trials [12-14]. Possible disease stages were clear/almost clear, mild/moderate non responders and severe non responders. The time frame was 22.4 years. Costs and clinical benefits were both discounted with 3.5%.

#### Average patient profile

Data from patients aged 18 to 75 years of age with severe CHE non-responding to standard therapy were included in the model. The exact patient profile was described in an earlier publication by Ruzicka et al [12]. The clinical study was registered with the U.S. National Institutes of Health (NCT00124475). Health status was determined according to the PGA criteria of clear/almost clear, mild/moderate and severe non responders [12]. A relapse for responding patients was defined as 'modified Total Lesion Symptom Score' (mTLSS) ≥75% of the original baseline value.

#### Treatment pathways

**Figure 1:**
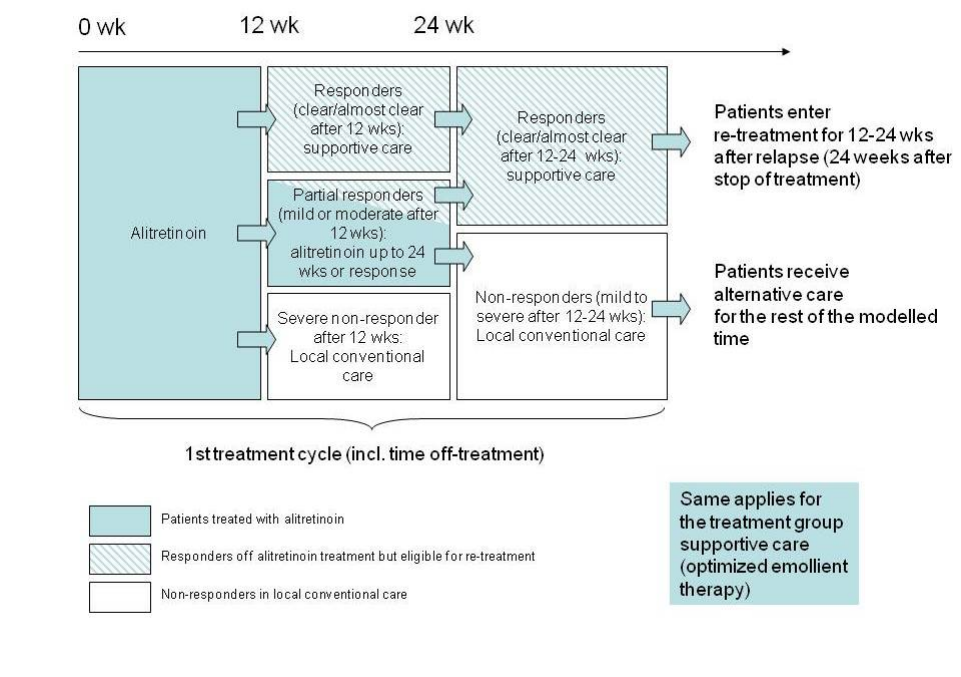
**Flow of model**.

The schematic model is presented in Figure 1. The two initial treatment strategies were the following: the alitretinoin group was treated with 30 mg once daily and the control group (supportive care) received optimised emollient care. Resource utilisation for both groups is described in Table 1. Treatment duration was 12 to 24 weeks per cycle, depending on response.

**Table 1:** Resource utilisation

Cost item	Costs per month (€)	Dosage per month	**Ref**.
*Basis costs for alitretinoin group*			

Alitretinoin 30 mg capsules	28 × €17.50 = € 490	28 × 30 mg (one capsule per day)	[19]
Emollients	€ 25	200gr	Official price in Swiss pharmacies
Pregnancy testing + oral contraceptives	€ 19	1 test, 21 tab	[19]
Dermatologist visits	€ 27	1 visit	[20]
Lipid monitoring tests^#^	€ 11	1 test	[44]
*Basis costs for placebo group*			

Emollients	€ 25	200gr	Official price in Swiss pharmacies
Dermatologist visits	€ 27	1 visit	[20]

*Following costs for non-responders (alitretinoin or supportive care): Conventional local therapy for severe non-responders*			

Emollients	€ 25	200gr	Official price in Swiss pharmacies
Dermatologist visits	€ 27	1 visit	[20]
PUVA/311 nm (Topical/oral)	€ 121	20 cycles per 10 weeks in 6 months period (3,33 cycles per month)	[20]
Topical steroids Class I-III	€ 30	60 gr	[19]
Topical steroids Class IV	€ 17	60 gr	[19]
Topical steroids total*	€ 20	60 gr	[19]

^#^LDL, HDL, Triglycerides.

*Assumes 25% of the patients treated with Class I-III and 75% with Class IV topical steroids

#### Transition probabilities

Transition probabilities were derived from information on response rates published by Ruzicka [12,13]. Response rates were assessed using the investigator's assessment (PGA scale).

Patients who responded (clear/almost clear) during the treatment cycle discontinued the therapy after 12 weeks at the earliest. Patients who remained in the severe state after 12 weeks stopped the treatment and entered conventional local treatment for severe non-responders. Non-responding or mild to moderate responding patients (after 12 weeks) were subject to receiving the therapy up to 24 weeks. Only patients responding (clear/almost clear) after the end of the first treatment cycle were eligible to re-enter further treatment cycles after relapse. In the period between treatment and relapse, these patients received maintenance therapy. The remaining patients (mild to moderate partial responders and severe non-responders) were treated with conventional local treatment for severe non-responders (Figure 1).

The average duration of treatment for responding patients receiving alitretinoin or supportive care was 15.43 weeks or 18.12 weeks for the first cycle and 14.77 weeks or 17.66 weeks for the re-treatment phase, respectively[14]. Time to relapse was assumed to be 24 weeks for both groups [13]. The alitretinoin and supportive care model was terminated after 29 and 27 re-treatment cycles, respectively. The total life span covered by the model was 22.4 years. Model outputs included costs and quality adjusted life years (QALYs) associated with each treatment strategy.

### Data source

Treatment usage was taken from the study protocol and reproduced by Swiss standards [12,18]. All resource estimations were discussed with a Swiss dermatologist and considered to be realistic and appropriate.

The relevant cost data, including alitretinoin, were derived from public prices as reported for the products by the Swiss pharmaceutical compendium [19]. Furthermore, the Tarmed (the official Swiss Tariff code) gave insights into the costs of medical services, diagnostics and therapies [20]. The private Laboratory Viollier, Basel, Switzerland, provided laboratory testing information. All tax points were converted into Euro (1TP = CHF0.92 = €0.613, January 2010).

#### Swiss epidemiology of CHE

The Central Office for Statistics in Accident Insurance (SSUV) from the Statistics institutions in Switzerland collects data on recognised occupational diseases and accidents since 1997 [21,22]. Swiss incidence rates and general costs of patients suffering from CHE were assessed from this basis.

#### Resource utilization and cost input for both treatment arms

Resource utilization included the amount of used medications per cycle, concomitant topical emollients, and rescue topical dermatological medication as well as psoralen and ultraviolet A radiation (PUVA). All patients were supposed to undergo a monthly dermatological visit, until a complete response was achieved. Patients reaching the status "clear/almost clear" were assumed to cut in half the number of dermatologist visits during the time period covered by the model. This assumption is in concordance with daily practice, where clear patients do not require visiting their dermatologist as frequently as patients in a severe state. All patients were given an emollient cream for daily usage.

#### Basis costs of alitretinoin group

Costs of basis treatment with alitretinoin included emollients without an active agent and one consultation monthly as shown in Table 1. The use of emollients was supposed to drop by 25% when "clear/almost clear" status reached. Due to the dose dependent effect of alitretinoin, patients received one 30 mg capsule daily [12,13]. All patients were treated until response was achieved. Laboratory tests were established to control and analyse triglycerides and cholesterols (low-density lipoprotein (LDL), high-density lipoprotein (HDL)). Women of childbearing age received after pregnancy tests oral contraceptives (0.15 mg Levonorgestrel, 30 μg Ethinylestradiol) before, during and after the treatment with the 9-cis retinoic acid.

#### Basis costs of supportive care group

Supportive care treatment for patients suffering from CHE composed basis emollients without active agents and one monthly dermatologist visit as described in Table 1.

#### Costs of following treatment

Responders received maintenance therapy for clear or almost clear patients, including emollients and dermatologist visits, as described above. Mild responders or severe non-responders were treated with conventional local care. This group obtained, besides the basic therapeutic interventions (emollients, consultations) psoralen and ultraviolet A radiation (PUVA) as well as applied topical steroids. The model assumed that twenty PUVA sessions were used during ten weeks in a six month period (2 sessions per week). Hence, the yearly average of PUVA therapy would sum up to 40 sessions. Per PUVA cycle 40 mg 8-methoxypsoralen were given (assumed body weight of 66-80 kilogram). PUVA baths were not taken into consideration as their usage is uncommon in Switzerland. Prednicarbonate (0.25%) and mometasone-17-furoate (0.1%) have moderate anti-inflammatory effects and were used as topical steroid class III. Clobetasol-proprionate (0.05%) was used for strong or very strong anti-inflammatory virulence (class IV) (Table 1).

#### Utilities

CHE may impair the qualities of life of the patient itself and of the patients' family. According to the National Institute for Health and Clinical Excellence (NICE) guidance document on adalimumab in psoriasis, severe psoriasis is defined as "a total Psoriasis Area Severity Index (PASI) of 10 or more and a Dermatology Life Quality Index (DLQI) of more than 10" [23]. Hence, CHE patients with a PGA status of severe, having a DLQI score greater than 10, can be compared to severe psoriasis patients in regard to the impact of the disease on their life-quality [24].

A 'mapping' exercise was carried out to estimate the association between psoriasis-related quality of life (QOL) (as measured by the DLQI) and utility (using the EQ-5D). The DLQI values were converted to EQ-5 D utility weights using the algorithm from Woolacott *et al *[25]. The following utility mapping formula was applied: EQ-5 D utility score = 0.956- [0.0248 × (DLQI total score)]] [25]. The underlying data of the DLQI scores by PGA severity was assessed from a study conducted by Freemantle et al [14,26]. This measurement of quality of life was also used in the recent NICE health economic appraisal of alitretinoin and assumed as adequate [27]. Utility values used in the model are visible in Table 2.

**Table 2:** Utility values in the model

	Clear/almost clear	Mild/moderate	Severe	Notes
DLQI Scores by PGA status	1.74	7.86	15.08	[26]
EQ-5 D Values*	0.913	0.761	0.625	[25]

*EQ-5 D utility score = 0.956 - [0.0248 × (DLQI total score)].

### Sensitivity analysis

In order to analyse the sensitivity of the model outcome, the most relevant clinical scenarios were considered in several univariate sensitivity analysis. The following key input parameters were taken in account: utilities related to all PGA states, supportive care costs, use of PUVA and efficacy of supportive care. To identify the most relevant input parameters, which influence the base case result, variations of ± 30% or ± 35% were applied.

## Results

### Cost-effectiveness analysis

#### Base-case analysis

After the first cycle, the cumulative response rate in the alitretinoin and the supportive care group was 47.7% and 16.6%, respectively [12]. The cumulative efficacy of clear/almost clear patients in the re-treatment cycles achieved 79.6% in the alitretinoin and 25.5% in the supportive care fraction[18].

The monthly drug costs of CHE patients are presented in Table 3. In the group of alitretinoin, costs summed up to €556, whereas the expenses of the supportive care treatment resulted in €52 per month. Patients with a clear or almost clear status accounted for €33 and the conventional local treatment of severe non responders was €193 (Table 3).

**Table 3:** Average costs (per month)

Costs item	Patients on Alitretinoin 30 mg*	Patients on Placebo^#^	Patients Clear or almost clear maintenance	Severe Non Responders^¥^
Dermatologist visits	€27	€27	€14	€27
Alitretinoin drug cost	€490	-	-	-
Emollients	€25	€25	€19	€25
Lipid monitoring	€11	-	-	-
Pregnancy testing and oral contraception°	€3	-	-	-
Alternative Care Non-Responders (PUVA + Topical Steroids)	-	-	-	€141
				

**Total**	**€556**	**€52**	**€33**	**€193**

°Female alitretinoin patients only. Assumes that 15% of female patients are in childbearing age and needing contraception.

*Including Mild/Moderate alitretinoin non-responder.

^#^Including Mild/Moderate placebo non responder.

^¥^Alitretinoin and placebo patients receiving conventional local care

During the entire modelling period, PUVA sessions per non-responding patient in the alitretinoin and the supportive care group resulted in 294.5 (according to 38.7 weeks on PUVA) and 295.9 (according to 41.7 weeks on PUVA), respectively. The total costs of a patient treated with alitretinoin or conventional treatment summed up to €42'208 or €38'795, respectively. Differences in effectiveness between the two therapies arose from the much higher response rate in the alitretinoin group compared to supportive care. Hence, patients receiving alitretinoin yielded on average a gain of 11.21 quality adjusted life years (QALYs). Patients in the supportive care group resulted in a lower efficacy (10.98 QALYs). Hence, alitretinoin therapy was associated with incremental costs of €3'413, accompanied with a gain of 0.23 QALYs per patient. Accordingly, the corresponding incremental cost-effectiveness ratio (ICER) was €14'816 per QALY gained, when compared with the control group.

Table 4 presents other Swiss cost-effectiveness studies. Alitretinoin treatment was cost-effective in comparison with existing cost-effectiveness thresholds.

**Table 4:** Examples of other Swiss cost-effectiveness analyses (CEA)

Study (Ref)	Indication	Comparison	Cost- effectiveness
[45]	CEA of quadrivalent HPV vaccine in girls aged 11 years over lifetime	Cytological screening vs. cytological screening with vaccination	€17'337/QALY*
[46]	CEA of eplerenone in patients with left ventricular dysfunction after myocardial infarction (EPHESUS study)	Eplerenone vs. placebo	€7'558/QALY*^1 ^to €15'977/QALY*^1^
[47]	Cost-effectiveness of trastuzumab in the adjuvant treatment of early breast cancer	Trastuzumab vs. standard care	€40'505/LYG^2^ €19'673/LYG^3^

*Currency conversion CHF1.00 = €1.5.

^1^depending on survival estimate.

^2^after 10 years.

^3^after 15 years.

#### Sensitivity analysis

**Figure 2:**
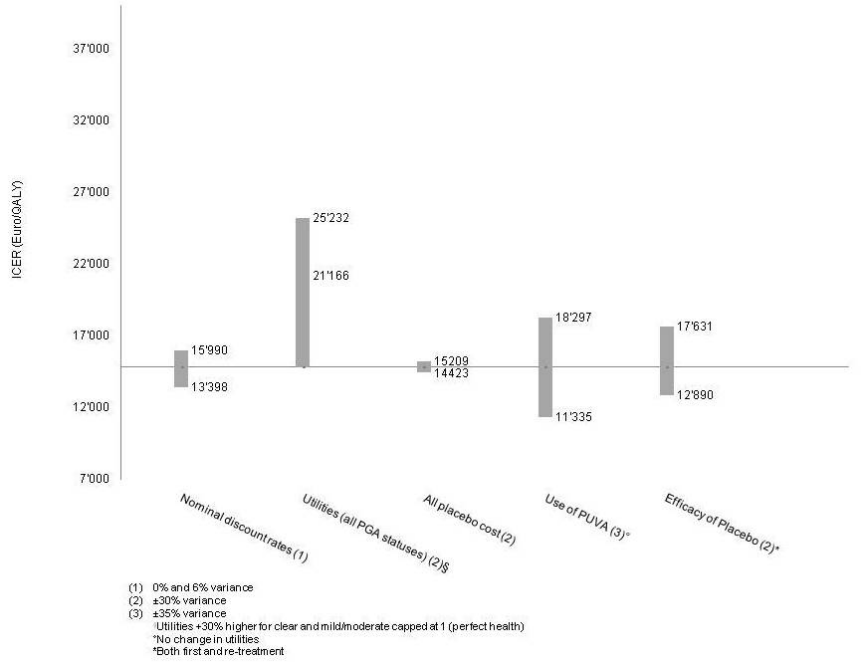
**Scenarios of sensitivity analysis**.

Several one-way sensitivity analyses were performed in order to guarantee the models' insensitiveness to particular parameter variations. For the analyses, model parameters were selected as described in the methods and were tested for their impact on the base case result. The impact of the scenario analyses (± 30% or ± 35% variation of selected parameters) on ICER results was minor (Figure 2).

### Burden of disease of CHE in Switzerland

According to the SSUV, 1147 cases of CHE patients were reported during the period between 1997 and 2006, whereof 91 patients were counted in the year 2006[21]. Consequently, the proportion of CHE in Switzerland ranges up to 2 per 100'000 habitants (total working population in 2006 (20-65 years of age): 4'319'266)[28]. These data are limited to work related cases covered by SSUV; hence, the incidence reported by the SSUV is underestimating the total number of Swiss CHE patients.

## Discussion and conclusion

Eczema of hands due to occupational activities is a common disease in industrialised countries. Treatment options for CHE patients are ample, but the efficacy is limited. Current existing therapies include topical or systemic treatments, avoidance of the exposed irritants by gloves, educational programmes and lifestyle changes [7,29-31]. Local phototherapy, and off-label use of retinoids, calcineurin inhibitors, immune suppressants such as systemic steroids and ciclosporin are alternative escalation steps for those who do not respond adequately to standard care including topical steroids [32].

The object of this study was to assess the economic impact of using alitretinoin treatment for patients with chronic hand eczema refractory to standard treatment. Using the effectiveness data from a published clinical trial and cost assumptions based on the Swiss health system, a model was built to measure the cost-effectiveness of alitretinoin versus supportive care in Swiss patients. Endpoints, treatment used in the two arms, duration of therapy and the follow up of the published multicenter trial (BACH trial) were exactly reproduced in the Swiss regimen [12,18].

As reported by Ruzicka et al, the effectiveness data used in our model reached in the alitretinoin group nearly three times higher response rates (defined as clear or almost clear hands) than in the supportive care treated patients [12]. During a treatment cycle, the monthly costs of alitretinoin therapy were put at €556 whereas supportive care accounted for €52. At the end of the entire simulation (22.4 years), the incremental cost-effectiveness ratio yielded in €14'816 per QALY gained. The ICER expressed robustness for all variable variations in the one-way sensitivity analysis.

The yearly medical average cost of alitretinoin with treatment durations of 12.9 weeks (time-to-response in BACH study: 1.4 cycles and 165 days relapse time) would result in €2'212 (incl. value added tax) per patient in Switzerland. Consequently, the daily treatment cost per year would sum up to approximately €6.06. This estimate can be put in perspective to systemic treatment of psoriasis patients indicating cost ranges from $1'197 (€957) to $27'577 (€21'866) per year [33]. The cost-effectiveness analysis presented above determined the ICER of alitretinoin treatment at €14'816 per QALY gained. Using this result on the overall Swiss situation, we would expect to invest additional €310'583 in alitretinoin treatment per year to acquire a further gain of 20.93 QALYs for all new CHE cases in Switzerland (adapted to the year 2006 with 91 Swiss CHE patients) [21]. This translates into total annual costs of €1'348'256 in alitretinoin treatment to obtain one QALY in each new CHE case in Switzerland.

Given that 3'736 occupational cases of illness were reported in Switzerland, skin diseases in general induced by hurtful substances correspond to 20.2% of those cases registered during 2006 [34]. Hence, 12.1% of all skin diseases recorded by the Swiss Accident Insurance Agency correspond to CHE [21]. Similar reports from the United States estimated the proportion of hand eczema cases in regard to working place damages at 15% [35].

Overall, there is only little evidence of the financial burden of chronic hand eczema patients in the published literature [36,37]. According to the SSUV, the expansion of the eczema on the entire hand, forearm and/or arm summed up to €3'428'260 in the year 2006. These costs included medical expenses, daily allowance, integrity reimbursement, other capital payment and net present values of the disability pension during the entire year. The compensated working days added up to €8'827 per year (2006) [22]. Thus, the once daily intake of alitretinoin not only appears to have a huge clinical and quality of life benefit, but also a pharmacoeconomic advantage by means of reducing the direct and indirect costs discussed above. Cost-effectiveness analyses are key factors of whether new treatment agents are funded or not [38]. According to the National Institute for Health and Clinical Excellence (NICE) the ICER for the acceptability threshold is determined between 20'000 GPB/QALY (€23'322/QALY) to 30'000 GPB/QALY (€34'983/QALY)[39]. Hence, the long-term result of our simulation model (€14'816/QALY) is in concordance with the guidelines of the NICE, which are generally accepted, including in Switzerland.

This model gives a good sense of the cost-effectiveness of alitretinoin versus supportive care. However, the analyses based on the model are subject to certain limitations. It has to be taken into account, that only direct costs and no indirect costs were incorporated. It should be noted that hand eczema patients are associated not only with high direct but also with indirect costs [40]. As an example for skin diseases, Verboom et al stated that high cost-of-illness was particularly found outside the health-care system, but variations across countries are substantial [41]. Given the much higher response rate of the alitretinoin group compared to the supportive care group, we could have expected lower indirect costs which would indirectly influence the ICER. Secondly, the findings of our work can be hardly compared with other studies as only few controlled clinical trials have provided adequate evidence-based data in regard to hand eczema refractory to standard treatment [42]. At the time of modelling, cost-effectiveness studies on the treatment of eczema are mostly lacking in the published literature [43]. Furthermore, we assumed constant efficacy (meaning transition probabilities) for the re-treatment cycle. The underlying clinical principals would presumably need some modification during the long-term treatment process. Given the available data source, this modification was not manageable in our analyses. Thirdly, our model pursued a conservative assumption supposing the same price of the drug over all cycles. However, over a long-term projection, the drug price of alitretinoin is likely to be amended due to price cuts or introduction of reference prices in some countries. Finally, the Swiss incidence numbers utilised in our economic evaluation correspond to work-related cases. Hence, the number and costs of hand eczema patients assessed by our work are likely to be underestimated.

The treatment of CHE patients in Switzerland is, according to expert opinion, insufficient as only few patients receive a clear status with the current treatment options. Due to the chronic course of the disease, the quality of life and social functioning often change for the worse. However, the new treatment option with alitretinoin raises hope for many patients. In our long time model we identified the therapy with alitretinoin as a cost-effective alternative for the therapy of CHE patients in Switzerland. The model used in our study may be valid not only in the Swiss setting but also in other European countries.

## Competing interests

This study was made possible by an unrestricted, educational grant of Basilea Pharmaceutica AG, Basel, Switzerland.

## Authors' contributions

PRB collected and assembled the data, performed the analysis, and wrote the manuscript. AAB contributed to the data assembly and data analysis. TDS designed the project, contributed to the data interpretation and supervised its development. All authors have read and approved the final manuscript.

## Pre-publication history

The pre-publication history for this paper can be accessed here:

http://www.biomedcentral.com/1471-5945/10/4/prepub
